# Optical Waveguide Lightmode Spectroscopic Techniques for Investigating Membrane-Bound Ion Channel Activities

**DOI:** 10.1371/journal.pone.0081398

**Published:** 2013-12-10

**Authors:** Inna Székács, Nóra Kaszás, Pál Gróf, Katalin Erdélyi, István Szendrő, Balázs Mihalik, Ágnes Pataki, Ferenc A. Antoni, Emilia Madarász

**Affiliations:** 1 Institute of Experimental Medicine, Hungarian Academy of Sciences, Budapest, Hungary; 2 Semmelweis University, Department of Biophysics and Radiation Biology, Budapest, Hungary; 3 MicroVacuum Ltd., Budapest, Hungary; 4 Egis Pharmaceuticals PLC, Budapest, Hungary; Biological Research Centre of the Hungarian Academy of Sciences, Hungary

## Abstract

Optical waveguide lightmode spectroscopic (OWLS) techniques were probed for monitoring ion permeation through channels incorporated into artificial lipid environment. A novel sensor set-up was developed by depositing liposomes or cell-derived membrane fragments onto hydrophilic polytetrafluoroethylene (PTFE) membrane. The fibrous material of PTFE membrane could entrap lipoid vesicles and the water-filled pores provided environment for the hydrophilic domains of lipid-embedded proteins. The sensor surface was kept clean from the lipid holder PTFE membrane by a water- and ion-permeable polyethylene terephthalate (PET) mesh. The sensor set-up was tested with egg yolk lecithin liposomes containing gramicidin ion channels and with cell-derived membrane fragments enriched in GABA-gated anion channels. The method allowed monitoring the move of Na^+^ and organic cations through gramicidin channels and detecting the Cl^–^-channel functions of the (α_5_β_2_γ_2_) GABA_A_ receptor in the presence or absence of GABA and the competitive GABA-blocker bicuculline.

## Introduction

Besides routine monitoring food quality, environmental pollution and health safety conditions, biosensors gain increasing significance in drug development, mainly in preclinical screening of novel pharmaceuticals. Biosensors, based on surface plasmon resonance [1] or optical waveguide lightmode spectroscopy (OWLS) [2], detect optical changes in a narrow field of evanescent light over the sensor surface. These label-free techniques provide real time information on molecular interactions including antigen–antibody or water-soluble receptor–ligand reactions. Many potent drug candidates, however, target membrane-embedded or membrane-associated proteins, which require appropriate lipid environment for preserving active conformation or assembling into functional molecular complexes. Several forms of artificial lipid environments have been built on sensor surfaces [3]–[8], including planar or supported lipid mono- and bilayers, and single or multi-layers of liposomes. Besides remarkable achievements, application of artificial lipid layers in sensor technology faces several difficulties. Incomplete continuity and mechanical vulnerability of the lipid layer(s) were shown to restrict reproducibility and decrease the life-time of lipid-functionalized sensors. In order to monitoring ion channel functions, we aimed to produce an optical sensor set-up, which can provide a stable lipid-environment for lipophilic parts and water-filled spaces for the hydrophilic chains of channel proteins, without impairing the sensitivity of optical detection by OWLS methods.

The principle of OWLS detection [9], [10] is that linearly polarized laser light is coupled into a thin planar waveguide layer by an optical diffraction grating [11], [12]. The angle of light incidence resulting in maximum coupling (incoupling angle) depends on the refractive indices of both the sensor chip and the material on the sensor surface. Varying the angle of incidence of the laser light, the incoupling angle can be determined with high accuracy, and therefore, the refractive index, thickness and coverage (or mass) of the material on the sensor surface can be calculated with high precision. OWLS signals provide information on optical changes in a small volume above the sensor corresponding to the penetration depth of the evanescent light into the sensor surface covering medium.

In principle, simultaneous opening or closing of ion channels can be detected by measuring changes in the refractive index caused by the drifts of the ionic composition of the sensor covering fluid layer. For this end, the thin detection layer should be separated from the larger volume of bulk electrolyte inside the cuvette, and ion permeation should be restricted to migration through ion channels located in the separating layer. Lipid layers with built-in ion channels can serve both, as boundaries between electrolyte-filled compartments and as selective ion transducers. In such a two-compartment model, a relatively slow drift in the ionic composition will be detected by OWLS assays, rather than the kinetics of trans-channel ion movement. In reality, such assays are often corrupted if the separating lipid layer is leaky, while producing continuous (non-leaky) supported lipid layer(s) with inbuilt ion channels is not an easy task [8], [13]. Genuine particulate two-compartment models are provided by liposomes and biomembrane-derived vesicles. By optical recording, however, the move of ions through the membrane of vesicles can be hardly separated from ion migration in the free solution, if lipid vesicles are included in the optical detection field.

The two-compartment sensing model can be improved if lipid layer(s) or vesicles are kept at a distance from the detection field. A “spacer” can be inserted for serving two purposes: (*i*) the spacer should support the formation of lipid membranes or attachment of lipid vesicles, thus, beside the “distance-keeping holder” function, it should serve as an ion-barrier except at sites of built-in ion channels. For this end, a good “spacer” should also provide environment for assembling functional ion channels in the spacer-supported lipid layer(s). (*ii*) The sensor-facing, bottom side of the spacer should hold out lipid material/vesicles from the detection field, while allowing passive migration of ions to the sensor surface. Multiple layers of polyelectrolytes [14], [15] can serve as spacers, and were shown to support the formation and long-term stability of continuous artificial lipid layers. The highly charged ionic environment, however, does not favor the formation of functional assembly/conformation of membrane-bound proteins. Teflon membranes, on the other hand, were reported to support the formation of artificial lipid bilayers with built-in functional ion channels [16].

We propose a novel membrane-sandwich sensor-design for OWLS assays by inserting commercially available filter membranes into the measuring cuvette. A relatively thick polytetrafluoroethylene (PTFE) membrane can be filled with lipids or lipid vesicles containing the ion channels to be investigated. The lipid-filled membrane creates a water and ion resistant insulating layer, which can sufficiently reduce the bulk permeation of electrolytes to the sensing area. The fibrous PTFE membrane with large virtual pore sizes can entrap bigger membrane fragments or intact/ruptured GUV, SUV liposomes, but can not completely stop the escape of small-size lipoid material. Deposition of traces of lipid material onto the sensor surface, however, can be prevented by inserting a further thin, water- and ion-permeable polyethylene terephthalate (PET) membrane. This highly hydrophilic, small pore-size membrane keeps the optical detection layer free from lipids but provides direct electrolyte contact with the fluid layer covering the sensor surface. The ionic composition and, consequently, the refractive index of the small-volume “sensing” fluid will change if enhanced amount of ions arrive in response to ion channel opening in the lipid-filled layer.

Fibrous PTFE membranes saturated with liposomes provided sufficient compartment insulation and supported the self-assembly of gramicidin channels. To extend the assays for pharmacologically important membrane-embedded ion channels, an important neurological/psychiatric drug target, a GABA-gated Cl^−^/HCO_3_^−^ channel [17] was to be investigated. Instead of artificial reconstruction of the multi-unit receptor assembly, biomembranes were prepared from HEK293*^GABAα5β2γ2^* cells expressing the α5, β2 and γ2 receptor subunits, and were filled into PTFE membranes. The sensor set-up allowed detecting the move of Cl^−^ ions through channels opened by GABA and reduction of ion flux in response to the specific channel blocker, bicuculline. The data indicated that the main pharmacological characteristics of the multi-unit protein channel complex were preserved and gave confidence for further development of compartmentalized “membrane-sandwich” OWLS sensor set-ups for *in vitro* pharmacological studies.

## Materials and Methods

### Materials

Chemicals of reagent grade were obtained from Sigma-Aldrich (Hungary), unless stated otherwise, and were used without further purification. Solutions were made with deionized distilled water (18.2 MΩ.cm at 25°C) and were filtered through 0.22 µm Millex®GP filter (Millipore, Hungary). Ethanolamine (Loba Chemie, Germany), methylamine and guanidine were used in hydrochloride forms. Cl^–^-channels were investigated using artificial cerebro-spinal fluid (ACSF) (mM: 145 NaCl, 3 KCl, 1 MgCl_2_, 2 CaCl_2_, 10 D-glucose, 10 HEPES; pH 7.4) or Cl^–^-free ACSF (mM: 140 Na-acetate, 5 KH_2_PO_4_, 0.8 MgSO_4_, 1.8 Ca-acetate, 10 D-glucose, 10 HEPES; pH 7.4).

Polyethylene terephthalate (PET) membrane (RoTrac®; thickness 23 µm, with regular pores of 50 nm diameter) was kindly donated by Oxyphen AG (Switzerland). Polytetrafluoroethylene (PTFE) membrane (LCR; thickness 140 µm, virtual pore diameter 450 nm) was obtained from Millipore (Hungary).

Human Embryonic Kidney (HEK293) cell line, expressing α5, β2 and γ2 subunits of human GABA_A_ receptors was established by researchers of EGIS Pharmaceutical Inc. (Hungary). Complete Mini Protease Inhibitor Cocktail Tablets were purchased from Roche (Hungary).

### OWLS Assays

Deposited mass, refractive indices, effective refractive indices and the thickness of deposited material-layer on the sensor surface were determined using an OWLS 110 instrument with OW 2400 grating coupler sensors and BioSense 2.6 software (MicroVacuum Ltd, Budapest, Hungary). OW 2400 grating coupler sensors consist of a 12 mm×8 mm glass substrate covered with a thin (175 nm) SiO_2_-TiO_2_ waveguide layer (refractive index: n_f_ = 1.77±0.03) with an 12 mm×2 mm optical grating (2400 lines/mm) (MicroVacuum Ltd, Budapest, Hungary) [12]. The optical grating incouples light of a He-Ne laser at a given resonance angle into the waveguide layer in which the light is propagated by total internal reflection, generating an evanescent field extending 100–200 nm from the surface. In OWLS assays, incoupling of the incident laser beam occurs at two well-defined angles of incidence: one for transverse electric (TE) and one for transverse magnetic (TM) mode. Rotating the cuvette with ±7 degrees, four characteristic photocurrent peaks (one TE and one TM peak on both the positive and negative sides) can be detected at the incoupling angles αTE and αTM. The smallest angle-step of the rotation is 2×10^−4^ degree, and the angular resolution of the measuring device is 4×10^−4^ degree. The resolution is further smoothed to 1×10^−4^ degree by peak-fitting function, which determines the degree-value at the centroid of the peak-delineated area. The accuracy of photocurrent detection is ±1.526 pA. With these parameters, a real sensitivity of d(NTM)/d(nc)∼0.128 could be achieved.

The glass sensor chips were placed on the sensor holder (MicroVacuum, Hungary) and tightened to its sealing O ring. The sensor holder formed a flow cell above the glass sensor with a volume of 12.1 µl. The sensor holder is made from biocompatible PEEK material, the O ring is made from Kalrez and the tubings are made from Teflon. The inlet tubing was connected to a sample injection system (MicroVacuum, Hungary) furnished with a Rheodyne Model 9725 injector valve.

All assays were carried out at continuous flow of a “base-buffer” selected according to the experimental design (see in the text), at a rate of 23 µl/min, at 22°C (±0.1°C) temperature. Each experiment was run with continuous recording of NTE and NTM OWLS signals. The base line resulted by running through the “base-buffer” was recorded, and optical changes were related to this base-line. Experimental solutions (test-solutions) were applied when the base-line was stabilized or returned after a previous treatment. Test-solutions (see in the text) were injected in 100 µl aliquots into the continuous buffer-stream through the injector valve. To accelerate detection velocity (10 data points/min), NTM and NTE data were collected from a range of ±0.2 degree around the incoupling angles.

### Determination of Optical Refractive Indices of Solutions

OW 2400 sensors inside the OWLS sensor holder were perfused with deionized water until the stabilization of the baseline. The refractive index and the thickness of the waveguide film (n_f_ and d_f_ respectively) were determined by the self-calibrating protocol of the instrument. Samples of solutions were injected into the cuvette and incoupling angles (αTE and αTM) were measured at λ = 632.8 nm wavelength. The effective refractive indices (NTE and NTM), and the air-related refractive indices for each covering medium (n_c_; Table 1) were determined by BioSense 2.6 software.

**Table 1:** Refractive indices of the applied solutions determined by OWLS assays in transverse magnetic (n_cTM_) mode.

	Refractive index n_cTM_^a^
Solution	T = 22°C; λ = 632.8 nm
H_2_O (MilliQ)	1.331125
D_2_O	1.327487
150 mM NaCl^b^	1.334233
150 mM ethanolamine.HCl^b^	1.334325
150 mM guanidine.HCl^b^	1.336020
150 mM methylamine.HCl^b^	1.336487
ACSF	1.335462
Cl^–^-free ACSF	1.336885

^a^^
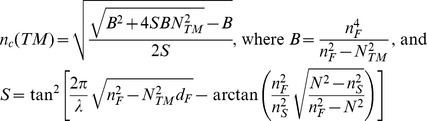
^.

Coefficients of variation were below 0.0003%.

^b^in MilliQ water buffered with 10 mM HEPES.

### Preparation of Lisosomes

Liposomes were prepared from egg yolk lecithin (composition: 70% phosphatidylcholine, 10% phosphatidylethanolamine and 20% other lipids including neutral lipids; Avanti Polar Lipids, USA) according to Moscho et al (1996) [18] (see Part S1.1 in File S1) in HEPES-buffered saline (HBS; composition in mM: 10 HEPES, 150 NaCl; pH 7.4) or in normal or Cl^–^-free artificial cerebro-spinal fluid (ACSF). The composition of ACSF in mM: 145 NaCl, 3 KCl, 1 MgCl_2_, 2 CaCl_2_, 10 D-glucose, 10 HEPES; pH 7.4. The composition of Cl^–^-free ACSF in mM: 140 Na-acetate, 5 KH_2_PO_4_, 0.8 MgSO_4_, 1.8 Ca-acetate, 10 D-glucose, 10 HEPES; pH 7.4.

Liposomes were occasionally labeled with Texas Red® DHPE (1,2-dihexadecanoyl-sn-glycero-3-phosphoethanolamine, triethylammonium salt; Invitrogen) and/or 1,2-dioleoyl-sn-glycero-3-phosphoethanolamine-N-biotinyl sodium salt (DOPE-biotin; Avanti Polar Lipids, USA). Texas Red labeled liposomes were used to check the lipid coverage of the holder membrane by Zeiss Axiovert 200M fluorescence microscope (see Part S1.2 in File S1).

### Preparation of Membrane-fractions from HEK293*^GABAAα5β2γ2^* Cells

HEK293 cells expressing human GABA_A_ receptors with α5 β2 and γ2 subunit composition (see Part S1.3 in File S1) were grown in Dulbecco’s modified Eagle medium, supplemented with 10% fetal bovine serum, 200 µg/ml zeocin and 3 µg/ml puromycin (as selection antibiotics), at 37°C, with 5% CO_2_. For membrane preparation, cells were washed off from the culture surface with 1 mM EDTA-PBS (pH 7.4). The cell suspension was distributed to 10^8^ cells/15 ml tubes and spun down at 200 g for 10 min. The cell-pellets were frozen and either stored or directly used for membrane preparation. Frozen pellet of 10^8^ cells was resuspended in 10-fold volume (200 µl) of ice-cold buffered saline containing protease inhibitors (applied according to manufacturer’s instruction). The cells were disrupted by 3 freezing-thawing cycles (dry ice for 2 min, 37°C water bath for 5 min), then the suspension was spun at 1100 g for 10 min at 4°C for removing larger cell debris and nuclei. The supernatant was centrifuged at 21000 g for 20 min at 4°C to sediment mitochondria. The 21000 g supernatant containing fragments of mixed cellular membranes was used in the OWLS assays.

### Functionalization of Sensor Surfaces with Multi-liposome Layers

Reactive amino groups were formed on the surface of sensor chips by treating with 10% (3-aminopropyl)triethoxysilane [19]. Biotin was coupled by incubating with 1 mg/ml NHS-biotin in phosphate buffer (mM: 358 Na_2_HPO_4_, 87 KH_2_PO_4_; pH 7.5) for 12 h at 4°C. After washing with deionized water, biotinylated sensors were inserted into the OWLS cuvette and washed with buffered saline until the stabilization of the baseline. NeutrAvidin (40 µg/ml) in buffered saline was injected into the cuvette, and washed out after 20 min incubation.

NeutrAvidin-functionalized sensor chips inside the OWLS cuvette were perfused with HBS. After stabilization of the baseline, consecutive layers of liposomes were bound to the surface according to the Membrane Protein Analysis Kit (Layerlab AB, Sweden) protocol. Briefly, 1.3 µM of biotin-ssDNA (15-base, single-stranded DNA with covalently bound biotin at one end) were injected onto the sensor surface. Meantime, liposomes were incubated (40 min; room temperature) with 1.3 µM Chol-dsDNA 1, (composed of one short and one long strand, with a cholesterol anchor at the paired termini of the chains), to gain approximately four (cholesterol anchored) DNA tags per liposome. The free floating single-stranded end of the long chain had a complementary sequence to the biotin-ssDNA bound on the biosensor surface. Chol-dsDNA-tagged liposomes were hybridized onto the biotin-ssDNA functionalized sensor surface. Multiple layers of liposomes were built by using another cholesterol-modified DNA (Chol-dsDNA 2) containing a single-stranded end identical in sequence to the biotin-ssDNA. Upon injection, Chol-dsDNA 2 tags were inserted into the membrane of liposomes on the sensor surface and a new layer of liposomes was formed by adding liposomes containing Chol-dsDNA 1 [21], [22]. Reagent excess was washed out by HBS prior to each injection.

### Insertion of Commercial Membranes into the OWLS Cuvette

Commercially available filter (PTFE as “holder” and PET as “separating”) membranes were cut to fit into the OWLS cuvette. A piece of PET separating membrane was placed on a sensor surface with carefully preventing ingress of air bubbles. Fibrous PTFE holder membrane pieces were soaked in the appropriate assay buffer or were functionalized with 20 µg/ml of NeutrAvidin. A piece of the PTFE membrane was layered above the PET separating membrane. The membranes were fastened with the flow-tight Kalrez O-ring of the OWLS sensor holder.

The inserted membranes occupied 2.1 µl from the total 12.1 µl cuvette volume. Proper insertion was checked after filling the PTFE membrane with lipid material (see below) by testing with a test-buffer with different refractive index in comparison to the running buffer. Incomplete insertion of the membranes or failure in lipid entrapment resulted in instant equilibration between the sensing volume and the running buffer. Such sensor set-ups were not (and could not be) used for assays.

### Application of Liposomes and Cell-derived Membrane Fractions onto the Holder Membrane

#### Egg yolk lecithine liposomes

A 100 µl aliquot of *egg yolk lecithine liposomes* corresponding 125 µg lecithin content was injected onto the PTFE membrane, which was already fitted on the sensor inside of the OWLS cuvette and was previously washed with the appropriate (HBS or ACSF, Cl^–^-free ACSF) assay-buffer. The inlet and outlet tubing was closed, and the suspension was let to sediment for at least 2 h. After sedimentation, the cuvette was streamed through with the assay buffer until stable NTM and NTE values against time were recorded by OWLS.

#### Cell-derived membrane fraction

A 100 µl aliquot of the 21000 g supernatant containing fragments of mixed cellular membranes of 5×10^7^ cells was injected into the OWLS cuvette, as above. After sedimentation, the cuvette was washed with Cl^–^-free ACSF until stable NTM and NTE values were measured.

#### Mixed liposome-biomembrane fraction

Mixed solutions of egg yolk liposomes and cell-derived membrane fractions were prepared by incubating 50 µl aliquot of the cell membrane fraction (derived from 2.5×10^7^ cells) with equal volume of liposomes (corresponding 62 µg lecithin) at room temperature for 2 h. 100 µl of mixed liposome-cell membrane suspension was injected into the OWLS cuvette as above, and was washed with Cl^–^-free ACSF until stable NTM and NTE values were measured.

### Application of Channel Building, Opening and Blocking Compounds

*Gramicidin D* stock solution was prepared in absolute ethanol at a concentration of 2 mg/ml, was further diluted with the base buffer (HBS) to contain gramicidin: lecithine = 1∶40 (1/40 molar ratio). Gramicidin solution was injected into the OWLS cuvette, which contained settled liposomes already assayed without gramicidin. After transfusion with 5× cuvette-volume (60 µl) of gramicidin-containing buffer, the flow was stopped and liposomes were incubated with the gramicidin solutions for 30 min, at 22°C. After the incubation, the gramicidin solution was washed out, and the OWLS assay was continued by recording the baseline and then the effects of consecutively injected solutions of channel permeating/blocking compounds.

*GABA* (γ-amino-butyric acid) and *bicuculline* were dissolved in (Cl^–^-containing) ACSF at a final concentration of 100 µM and were applied on sensor set-ups carrying mixed (liposome and cell-derived) lipid-membrane fractions equilibrated with ACSF. The test-solutions were injected into the stream of ACSF consecutively: 100 µl of Cl^–^-containing ACSF, 100 µl of Cl^–^-containing ACSF with 100 µM GABA, and 100 µl of Cl^–^-containing ACSF with 100 µM GABA and 100 µM bicuculline. Each injection was started after the return of the original NTM and NTE baseline.

### Determination of Assay-sensitivity

Sensitivity of the assays was characterized by determining the smallest detectable compound (ethanolamine, guanidine or Cl^−^) concentration, which resulted in measurable shift of the effective refractive indices (NTE and NTM). Concentration-series of compounds from 0 to 150 mM were prepared in the appropriate assay-buffers, and 100 µl aliquots were injected into a continuous assay-buffer flow under permanent NTM and NTE. Any effect was regarded detectable, if the evoked NTM and NTE changes exceeded the background (NTE and NTM at 0 compound concentration) with a three-fold value of the standard deviation of the background average (limit of detection; LOD, [20]). LOD values were determined for bare sensors and also for sensors furnished with empty PTFE and PET membranes or with membranes filled with liposomes and/or cell-derived biomembranes. For comparing data obtained on individual sensors, relative values of effective refractive indices (NTM or NTE) were calculated as percentages of the effective refractive indices measured at the highest analyte concentration (100%) in a given assay.

### Data Evaluation

Averages and standard deviations were calculated from data obtained from n≥4 independent series of identical experiments. In order to compare the results obtained on individual sensor-chips, either the absolute values of n_c_ changes (Δn_c_) or the relative values of effective refractive indices (NTM or NTE) (in percentages) were calculated. Significances were calculated by student *t*-test. Results with p<0.005 were accepted as significant.

## Results

Various OWLS sensor-arrangements were probed in order to find techniques for assaying ion permeation through self-assembling gramicidin cation channels and a multi-subunit anion channel, the GABA_A_ receptor. As optical grating coupler sensors respond to refractive index changes, pairs of buffer solutions were prepared by replacing one of their ionic constituents for a compound with different refractive index but comparable transmembrane permeation capability (Table 1). Besides proposing a novel membrane-sandwich sensor set-up, we present and discuss some less successful sensor-designs (see also Part S1.4 in File S1).

### Assays on Multilayers of Coupled Liposomes

**Figure 1:**
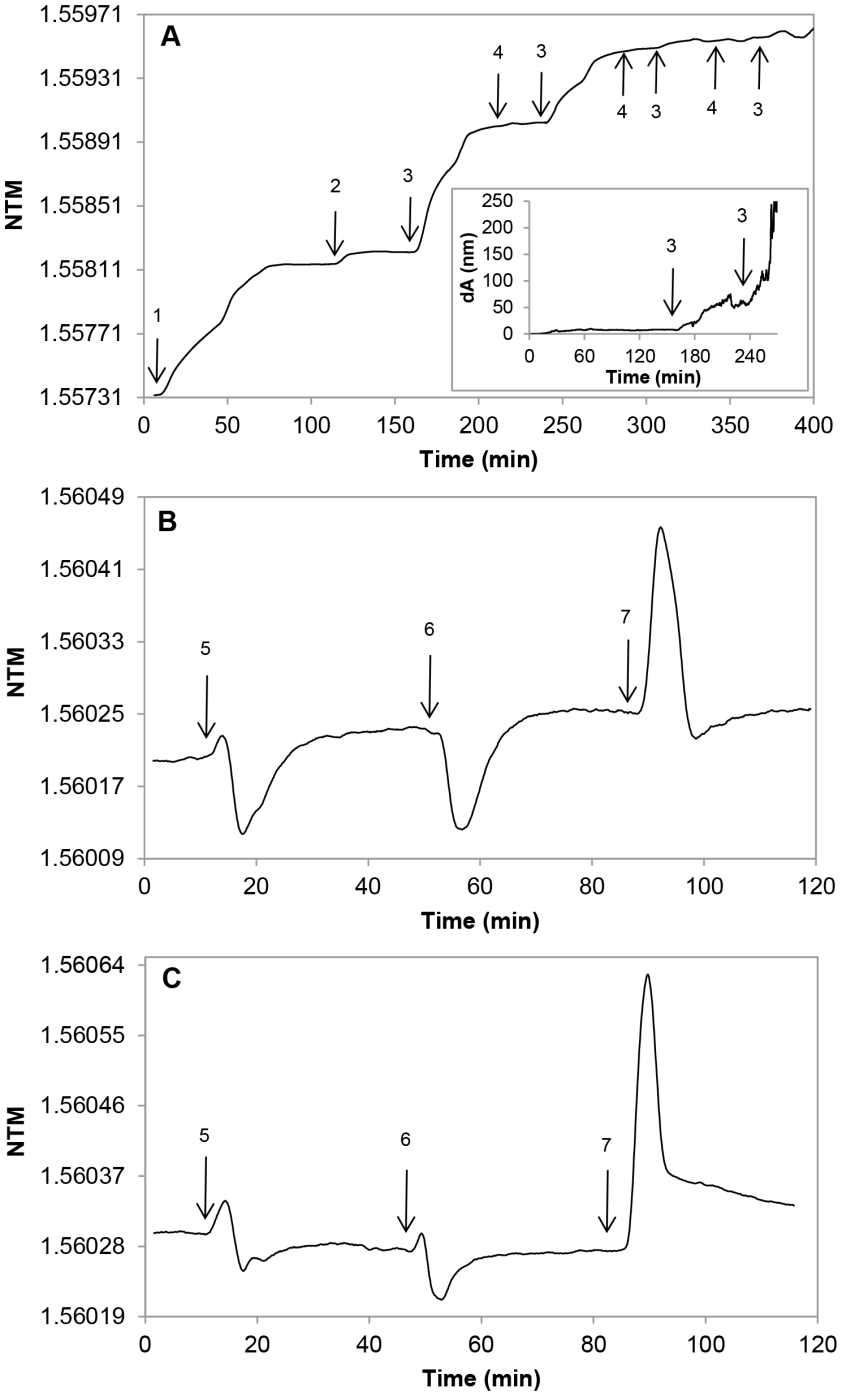
OWLS recordings of deposition of multiple liposome layers and permeability for small organic cations. (**A**) Biotinylated sensors were treated with (1) NeutrAvidin; (2) biotin-ssDNA; (3) Chol-dsDNA1-tagged liposomes; (4) Chol-dsDNA2. The insert shows the increasing thickness (dA) of the deposited material on the surface reaching the detectable maximum (∼200 nm) after the second injection of liposomes. (**B**, **C**) Changes of the effective refractive indices (NTM) in a representative series of experiments with cross-linked multilayer of liposomes without (**B**) or with (**C**) gramicidin channels after injections of ethanolamine (5), methylamine (6) or guanidine (7) solutions.

As it was shown by Branden and colleagues [21], [22], several properties of trans-membrane transport proteins embedded into liposome membranes can be investigated with evanescent optical (SPR) detection if concentration-drifts of slowly permeating analytes are monitored in the sensing volume. As an approach, we assembled multiple layers of interconnected liposomes [21], [22] on NeutrAvidin-functionalized sensor surfaces. Consecutive layers of liposomes were bound by DNA hybridization (Membrane Protein Analysis Kit). Through two subsequent hybridization steps, a thick (≥200 nm) layer of liposomes was built on the sensor surface (Figure 1 A), apparently exceeding the thickness of the optical detection field. Liposomes were clearly seen on sensors at the end of long-term OWLS assays by fluorescence microscopy on sensors carrying Texas Red labeled liposomes (see Part S1.5 in File S1). The observations indicated that interconnected liposome multilayers could be established and preserved on the sensor surface.

The interconnected layers of liposomes hydrated in HBS were probed to study the cation exchange through the lipid membranes, with or without inbuilt gramicidin channels. For Na^+^-free solutions, Na^+^ was replaced by small organic cations of ethanolamine, guanidine, and occasionally methylamine. Ethanolamine and methylamine are known to pass through gramicidin channels by different kinetics due to their different size and molecular shape, but do not block the channel [23]. In contrast, guanidine was shown to block the channels in artificial membranes by interacting with gramicidin protein residues and causing flicker blocks when passing through the channel [23].

Once the liposomes were assembled and the baseline in HBS was stabilized, 100 µl of Na^+^-free solutions of ethanolamine, methylamine or guanidine (150 mM buffered with 10 mM HEPES) were introduced into the continuous HBS flow, through the injector system. Each injection was followed by washing with HBS, and next test-aliquots were injected when NTE and NTM values at HBS washing returned to the base-line (Figure 1 B). After three injection cycles, gramicidin was incorporated into the liposome membranes and the assays with Na^+^-free solutions were repeated (Figure 1 C), in three consecutive series.

Injection of Na^+^-free ethanolamine or guanidine solutions (Figure1 B) caused an immediate increase in the NTM values indicating the arrival of a fraction of organic material (with higher refractive indices) to the sensor surface *via* free diffusion through the extra-liposome space. The initial rise in NTM increased in the presence of gramicidin (Figure 1 C), suggesting that a proportion of organic ions migrated through the channels and invaded intra-liposome volumes adjacent to the sensor surface. The subsequent decrease in NTM was reasoned as a mixed effect of rapid diffusion of Na^+^ ions out from the intra- and inter-liposome spaces in response to Na^+^-free perfusion, and a slower diffusion of organic compounds into the liposomes. In the presence of gramicidin, ethanolamine and methylamine molecules compensated the NTM decrease by entering liposomes, and thus, conquering a larger volume in the detection field. The assumption was supported by the effect of guanidine, which, by blocking gramicidin channels, was excluded from the liposomes and could partially block also the outmigration of Na^+^ from liposomes. As an additional effect, shrinkage of the liposomes in response to unbalanced inward and outward ion migration [22] can not be excluded.

While the recorded data including the robustness and reproducibility of the multi-liposome layer and the clear-cut differences between the exchange of ethanolamine and guanidine indicated that such approaches might be used for defined purposes, the time-resolution of OWLS detection did not allow analyzing the kinetics of permeation. Moreover, detection of concentration changes was hindered by summarized recording of optical changes from the entire detection field. With this sensor set-up, changes in the intra- or extra-liposome compartments could not be distinguished. The observations indicated that for getting interpretable OWLS data on transmembrane ion permeation, the different compartments should be either theoretically distinguished or physically separated.

### Assays with Membrane-sandwich Sensor Set-ups

**Figure 2:**
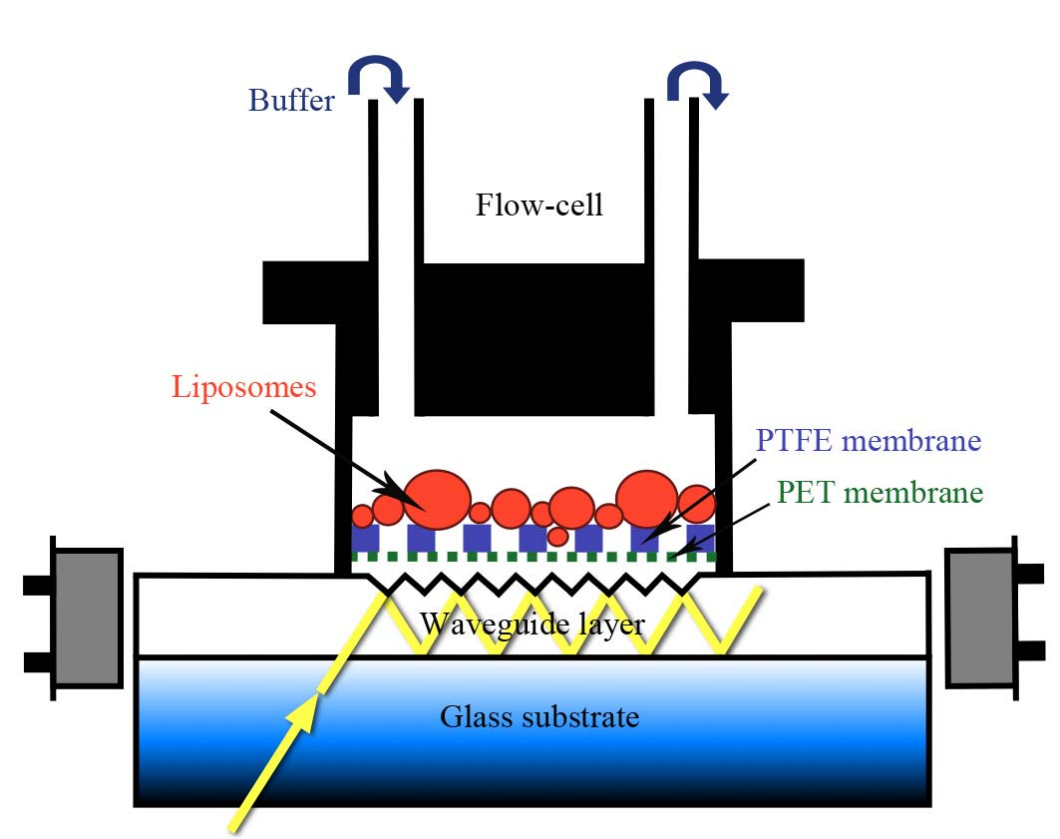
Schematic view of the membrane-sandwich sensor set-up. A thick (140 µm) PTFE lipid-holder membrane was placed on the top of a thin (23 µm) PET membrane for complete separation of lipid material from the sensor surface.

As a next approach, we excluded liposomes from the optical detection field by inserting a “spacer” between the sensor surface and the lipid-containing layer. We probed commercially available membrane filters as both, lipid holding supports and compartment separating sheets. Two different filter-membranes were used: (*i*) a fibrous PTFE membrane was applied as a lipid-holder layer and (*ii*) a PET membrane with uniform, straight cylindrical capillary pores was inserted to protect the sensor surface from lipid material. The PET membrane was fitted onto the sensor area and the lipid-holder PTFE membrane, with or without functionalization, was layered above it (Figure 2).

Layering the empty “holder” and “separator” membranes onto the sensor reduced the photocurrent peaks, but did not displace significantly or widen the incoupling peaks. Changes in the amplitude of the photocurrent peaks do not disturb the OWLS assays as long as the peak-position can be precisely determined. The small changes in the peak-positions, however, indicated that the separator PET membrane occupied a small fraction of the sensing volume and thus, physically decreased it.

**Figure 3:**
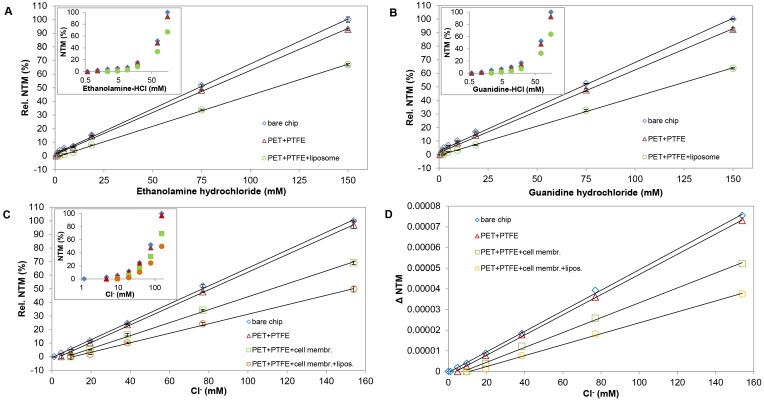
Sensitivity of different sensor set-ups to selected ions. Relative NTM values were determined as functions of ethanolamine (**A**), guanidine (**B**) and Cl^−^ (**C, D**) concentrations using different sensor set-ups including bare sensors and sensors furnished with empty PET+PTFE membranes, PET+PTFE membranes+liposomes, PET+PTFE membranes filled with cell-derived biomembranes and PET+PTFE membranes filled with cell-derived biomembranes+liposomes. Relative NTM (%) were calculated as percentages of NTM values detected at the highest (150 mM) compound concentration (100%) for each sensor set-up. The inserts show representative series of data in semi-logarithmic plots for give a higher resolution at low concentrations.

To characterize the sensitivity of the membrane-compartmentalized sensor set-up, NTM values were measured in response to solutions with different concentrations of selected ions on bare sensors and on sensor set-ups furnished with empty and lipid-filled membranes (Figure 3). NTM in a base-buffer and in base-buffer with different concentrations of a test compound were compared to NTM measured at the highest applied concentration (150 mM) of a test compound (100%). The relative values of effective refractive index-changes were comparable among the different sensor set-ups (Figure 3).

As OWLS detects refractive index changes in the range of 10^−6^ n_c_ values, very small alterations in the composition of the sensor-covering fluid layer generate measurable signals. To distinguish signals from noise, NTM values for each running buffer were carefully analyzed and the average NTM with standard deviations were determined for each sensor set-up in each base-buffer. NTM-changes to test solutions were accepted as specific optical responses, if the NTM value in test-solution exceeded the NTM in base-buffer with a three-fold value of the standard deviation [20].

On bare chips, the compound concentration on the sensor surface was regarded equal to the concentration of the bulk solution in the OWLS cuvette, so the detection limit could be determined by measuring the effective refractive index (NTM) as a function of the concentration of the injected fluid (Figure 3). The assays with bare sensors detected 0.72 mM concentration of ethanolamine, 0.75 mM concentration of guanidine and 2.91 mM of Cl^−^ (LOD values) corresponding to 1.38×10^12^ ethanolamine molecules, 1.44×10^12^ guanidine molecules or 5.59×10^12^ of Cl^−^ ions in the sensing volume (V = width×length×height of evanescent field = (2000×8000×0.2) µm^3^ = 3.2 nl). After introducing the PET and PTFE membranes, the detection limits increased, and the apparent sensitivity was further reduced if the PTFE holder membrane was filled with lipid material (Table 2). The data, however, does not mean necessarily that the sensor sensitivity was impaired; rather, it may indicate the rate of compound retardation by the inserted membranes. As we could not clear up this point, the smallest detectable concentrations recorded in empty PET+PTFE filter containing set-ups were regarded as the LOD in the assays with membrane-sandwich set-ups.

**Table 2:** The minimum amount of detectable material in assays with various sensor set-ups.

	Concentration in the injected buffer [mM]			Number of molecules/ions in the sensing volume (3.2 nl)		
Sensor	ethanolamine	guanidine	Cl^−^	ethanolamine	guanidine	Cl^−^
bare	0.72	0.75	2.91	1.38×10^12^	1.44×10^12^	5.59×10^12^
PET+PTFE	0.82	0.89	9.64	*1.57×10^12^*	*1.75×10^12^*	*1.85×10^13^*
PET+PTFE+lipid	4.9	3.52	19.78	*9.41×10^12^*	*6.76×10^12^*	*3.8×10^13^*

All assays were run under permanent buffer perfusion at a rate of 23 µl/min, with injecting of 100 µl volumes of analyte solutions into the stream. Accordingly, the analytes were streamed through the cuvette in 4.35 min. The total washing-through period, however was expanded presumably by fluid-mixing and retardation resulting in a total washing-through period of about 10 min. After this period, the buffer-base line returned indicating the reversibility of the assays, regardless of the injected analyte.

### Detection of Cation Migration through Gramicidin Channels

**Figure 4:**
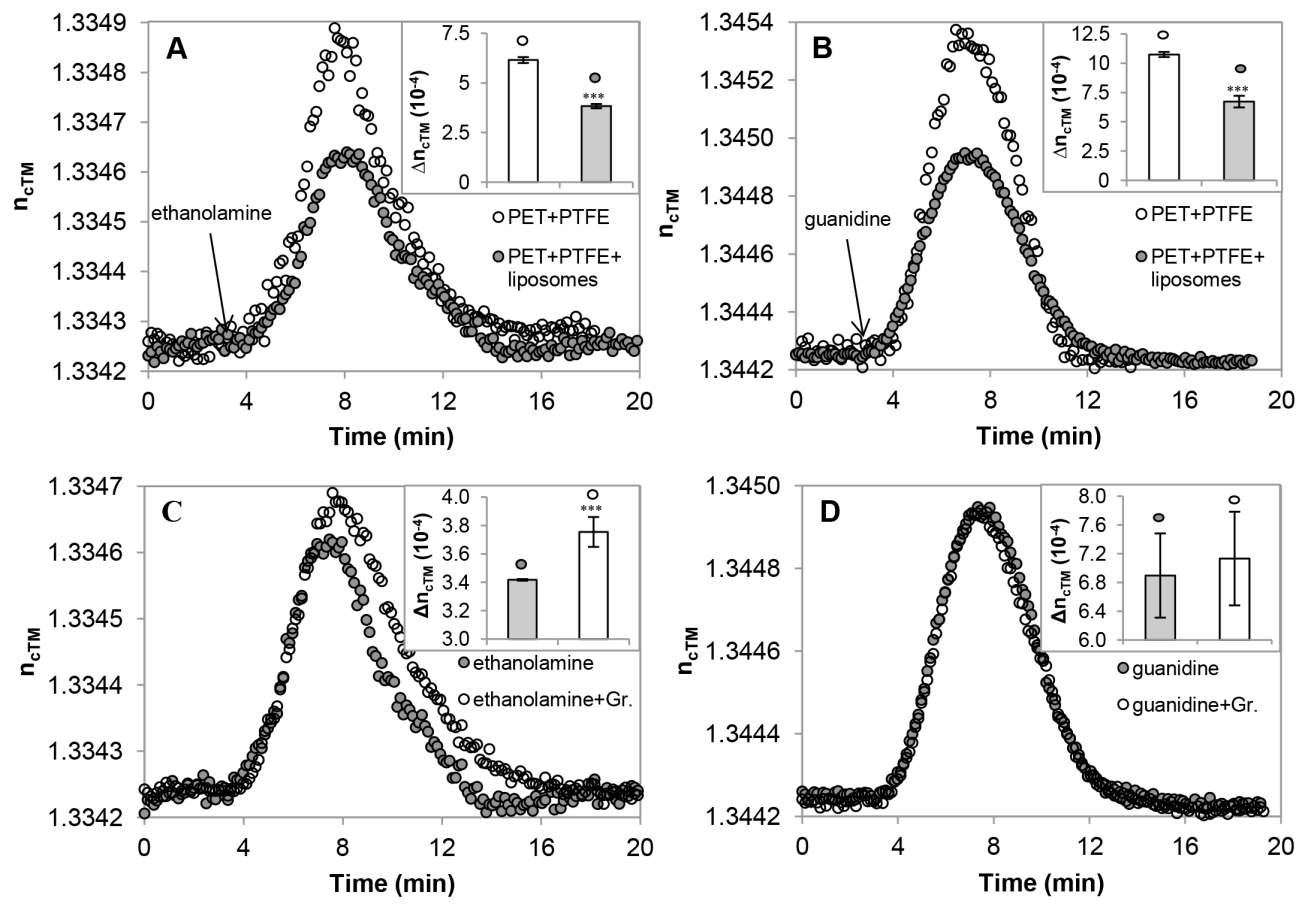
Detection of cation migration through gramicidin channels in membrane-sandwich sensor set-ups. Refractive indices (n_cTM_) of the sensor-covering fluid layer were measured in the transverse magnetic (TM) mode in sensor set-ups containing empty or lipid-filled filter membranes (**A**, **B**) after injecting ethanolamine (**A**) or guanidine (**B**) solutions, and after the incorporation of gramicidin (**C**, **D**). The inserts show averages and standard deviations of n_cTM_ changes, calculated from three independent experiments. Significant (***p<0.001; paired *t*-test) differences were found in response to filling with liposomes regardless of the chemical nature of the analyte (**A**, **B**). Incorporation of gramicidin, while resulted in enhanced permeation of ethanolamine (**C**), did not cause detectable changes in the move of guanidine (**D**).

The membrane-sandwich sensor set-up was probed for detecting exchange of Na^+^ for organic cations ethanolamine and guanidine, first through empty filter layers (PET+PTFE), then through membranes filled with liposomes (more exactly, liposome-derived lipid material) (PET+PTFE+liposomes), and finally through supported lipid material with inbuilt gramicidin channels (PET+PTFE+liposomes+gramicidin) (Figure 4). In each case, the set-up was equilibrated with HBS until reaching a stable baseline, then 100 µl aliquots of ethanolamine hydrochloride or guanidine hydrochloride (150 mM buffered with 10 mM HEPES, pH 7.4) were injected.

Lipid-functionalization significantly reduced the arrival of the injected solutes to the sensor surface, even if it did not separate the compartments completely (Figure 3 A and B, Figure 4). After three consecutive injections of ethanolamine or guanidine solutions, gramicidin channels were built into the lipid material, and the assays were repeated. In the presence of gramicidin, ethanolamine injection resulted in an increase of the refractive index (n_c_) indicating the enhanced influx of ethanolamine into the sensing volume (Figure 4 C). In contrast, the effect of guanidine was not influenced by the presence of gramicidin channels (Figure 4 D) in agreement with the known channel-blocking effect of guanidine. The membrane-sandwich set-up was used also to study the outward diffusion of ethanolamine from and the inward move of Na^+^ into liposomes hydrated in ethanolamine-Tris buffer (see Part S1.6 in File S1). The reproducible acceleration of the ethanolamine clearance in the presence of gramicidin verified the feasibility of the set-up, and let us to probe it with more realistic biomembrane vesicles and pharmacologically important channels.

### Assays on Cell-derived Membranes Carrying GABA_A_ Anion Channels

Cellular membrane fractions were isolated from HEK293 cells expressing human GABA_A_ receptors with α5, β2 and γ2 subunit composition. A crude membrane fraction (21000 g supernatant) containing fragments of mixed cellular membranes, was deposited onto the PTFE holder membrane alone or – in order to increase the lipid-saturation of the holder membrane – together with liposomes.

Different membrane-sandwich assemblies were assayed including empty PTFE and PET filter membranes, filter membranes filled with GABA-receptor containing cell-derived membranes and filter membranes filled with the cell-derived membranes together with liposome-derived lipids. The ion-barrier functions of different membrane-sandwich set-ups were monitored without adding any channel influencing compounds. The assemblies were perfused with Cl^–^-free ACSF until reaching a stable baseline, then 100 µl ACSF containing different concentrations of Cl^−^ was streaming through. Significantly less Cl^−^ ions arrived to the sensing volume if cell-derived membranes were added to the filters and even less Cl^−^ ions were detected if liposomes were added to biomembranes on the holder membrane.

**Figure 5:**
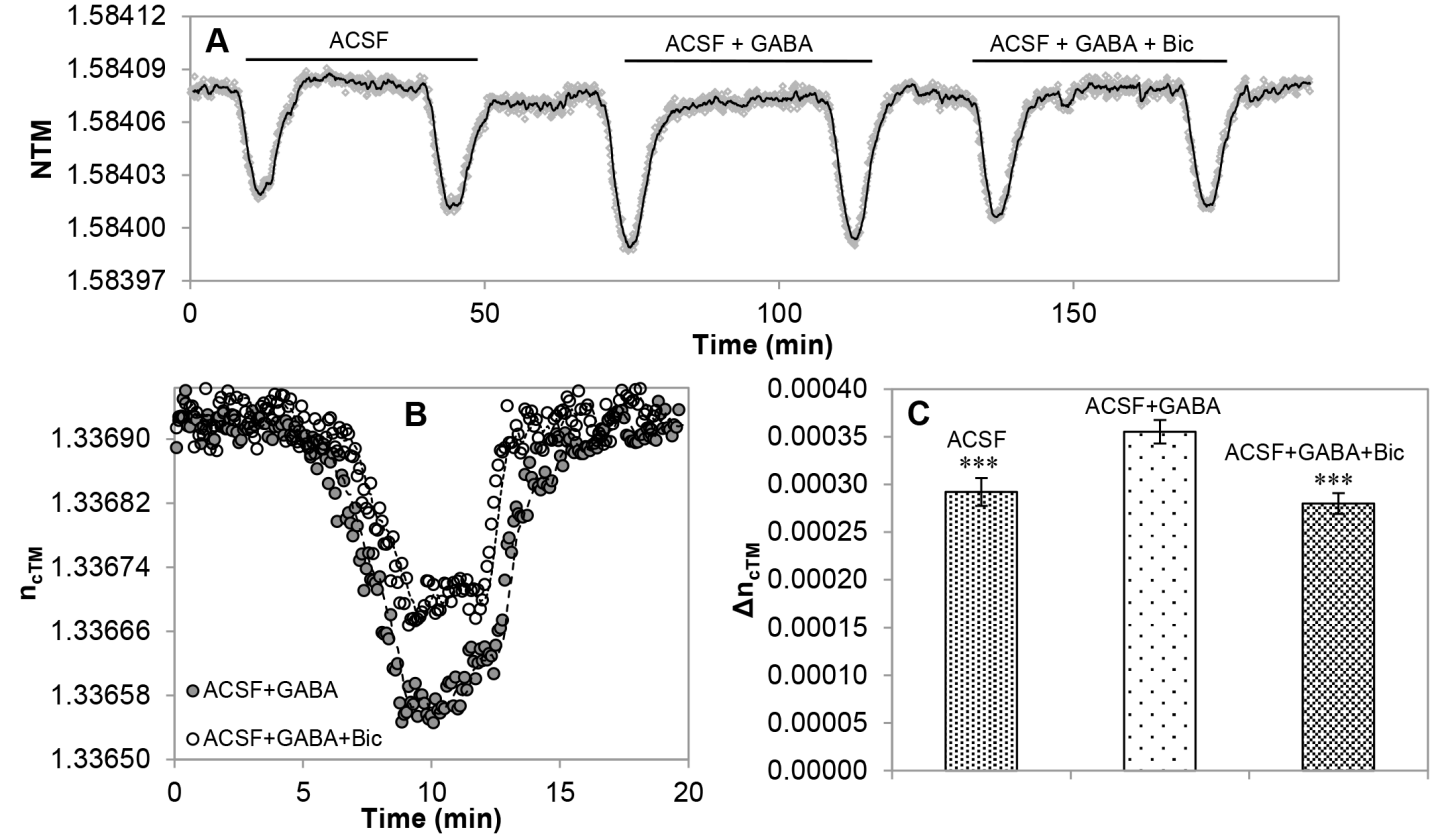
Demonstration of Cl^–^-channel functions of cell-derived GABA receptors in the membrane-sandwich sensor set-up. OWLS recordings were made in Cl^–^-free ACSF as running buffer with injection of Cl^–^-containing ACSF (ACSF) with or without GABA and the channel blocker bicuculline (ACSF+GABA and ACSF+GABA+Bic, respectively). (**A**) Changes of the effective refractive index (NTM) values in a representative OWLS assay. (**B**) Changes in the refractive index (n_cTM_) in response to the GABA-channel blocker bicuculline are shown from a representative experiment. (**C**) Summary of refractive index (nc_TM_) changes (Δnc_TM_) in response to transfusion with Cl^–^-containing buffer (ACSF), in the presence of the agonist GABA (ACSF+GABA) or in the presence of both GABA and the channel-blocker bicucullin (ACSF+GABA+bicucullin). Δnc_TM_ values were calculated from data of 4 independent series of experiments (n≥4); averages and standard deviations are presented.

In Cl^–^-containing ACSF, the decrease in NTM indicated the improper insulation of fluid-compartments (Figure 5 A). Injecting Cl^–^-containing ACSF together with GABA (100 µM), however, further decreased the refractive indices indicating an enhanced influx of Cl^−^ ions into the sensing volume in comparison to the effect of Cl^–^-ACSF alone (Figure 5 A and C). The Cl^–^-permeation was markedly reduced, if the solution contained also bicuculline, a potent GABA-channel inhibitor (Figure 5 A, B and C).

The data demonstrated that GABA channels preserved their basic function and drug-sensitivity in the membrane-sandwich. The finding indicates that the sensor set-up might provide an optical assay tool for studying lipid-associated, multi-subunit channels with pharmacological interest.

## Discussion

OWLS techniques detect changes in the refractive indices, caused by compositional and/or conformational changes in a narrow (200 nm) layer above the sensor surface. Changes in the functionalization of the sensor surface, including motion, shrinkage, swelling, fusion or detachment of lipid layers/liposomes, however, can also generate large changes in refractive indices, imposing serious limitations in recording molecular events. Moreover, due to the superposition of distinct optical events, a single data will be obtained at each detected time-point, regardless of the origin of the optical changes. With the presented data we wish to demonstrate that functionality and drug-sensitivity of trans-membrane ion channels can be investigated by OWLS assays, if concentration-drifts are monitored in a small sensing volume on the sensor surface. The optical data obtained with sensor-attached poly-liposome arrays demonstrated that for this purpose, the sensing fluid-space has to be separated from both intra-liposome and free buffer spaces. While coupled poly-liposomes were used to investigate membrane embedded channels with evanescent optical detection methods [22], our data obtained with this system led us to develop compartmentalized OWLS cuvettes, which allowed sufficient separation of the sensing volume from the rest of the assay system.

The proposed simple membrane-sandwich sensor set-up seems to overcome several shortcomings and might be worth for further development to obtain easily available methods for label-free optical detection of membrane-associated ion channel functions. By using commercially available membrane filters as lipid holders and compartment separating devices, the model allows creating artificial lipid environment for transmembrane proteins without troublesome creation of continuous lipid layers on solid supports. The fibrous inner structure of the PTFE filter allows infiltrating some larger fragments of biomembranes including vesicles or sheets of cell surface membrane. The holder membrane, even with a large (450 nm) virtual pore size, could withhold mixed sized lipid vesicles. For small liposomes, however, membrane filters with smaller virtual pore size, as those used for molecular filtration are worth for trying. The escape of liposomes or biomembrane fragments from the holder membrane and their contact with the sensor surface can be prevented by inserting a second membrane sheet with small regular, water- and ion-permeable pores.

In the OWLS detection system, prevention of optical disturbance is an important issue. The thin separating PET membrane reduced the intensity of the incoupled light as it was indicated by the reduction of the photocurrent peaks, but it did not destroyed the assay. A small but real shift in the position of the incoupling peaks, however, indicated that the membrane may invade a small layer from the evanescent field. The size of the regular pores (50 nm) in the PET membrane, however, was smaller than the size of inhomogeneities, which may cause significant widening of the incoupling angles [24], [25]. The preserved sharp photocurrent peaks indicated the robustness of the OWLS assay principle based on recording the optimum incidence angle of the laser light instead of detecting light intensity.

Ion channel proteins are usually applied in purified form for insertion into liposomes or artificial lipid layers. Trans-membrane channel proteins, however, easily lose native structure and activity during purification and reconstitution processes, especially if the active molecular complex consists of several subunits. In contrast to self-assembling ionophore proteins [26] as gramicidin or mellitin, safe methods are hardly available for inserting functional multi-subunit transmembrane proteins into artificial lipid environment. As an alternative, native cell-derived membrane fractions, those enriched genetically in a given transmembrane channel can be used. In our membrane-sandwich set-up, the irregular fibrous inner structure of the PTFE holder membrane provided functional environment for native biomembrane-embedded GABA_A_ receptors by preserving both the drug-sensitive hydrophilic and the membrane-embedded ion-conducting parts of the multi-unit molecular complex.

According to fluorescent microscopic analyses, the lipid material deeply invaded the fibrous filter-texture. Detailed data on the arrangement and proportion of compounds inside the fibrous filter-texture, however, can be hardly presented. We do not know whether the liposomes were (or in which proportion were) disrupted, in what extent get fused with the biomembrane material and what sort of size-separation might take place along the depth of the PTFE filter membrane. This uncertainty can be overcome by standardizing the loading procedure. In order to keep the sensor surface free from lipid material, the lipid-soaked holder membrane was separated from the sensor surface by a highly hydrophilic, small pore-size but water- and ion permeable separating sheet.

While the speed of sampling (6–10 detection/min) in OWLS assays is too slow to follow single channel changes, the compartmentalized “sandwich” sensor arrangement allowed detecting the drifts of the ionic composition in a thin fluid layer covering the sensor surface. The large concentration gradient (150 mM/163 µm given by the 140 µm and 23 µm thickness of the PTFE and PET membranes, respectively) assured directed migration of ions either to or from the small sensing volume. The OWLS assay proved to be sensitive enough to detect refractive index changes caused by 10^12^–10^13^ ions arriving to or escape from the thin sensor covering fluid layer. This amount of ions can permeate through about 1000 gramicidin channels and about 10000 GABA-gated Cl^−^ channels with average “open-state” single channel ion permeabilities of 10^7^ ion/sec [26] and 2.4×10^6^ ion/sec [27], respectively. While the number of incorporated channels was not precisely determined, the amount of introduced gramicidin proteins and the mass of biomembrane fraction prepared from 5×10^7^ cells each carrying immunocytochemically detectable amount of GABA receptors guaranteed enough channels for detection. Optically detectable channel-permeating ions were provided for a limited period lasting from the arrival of the injected fluid front to its complete washing out. During the washing-through period, drugs influencing GABA-channels acted as it was expected: the channel opening GABA increased, while the channel blocker bicuculline reduced the Cl^−^ concentration in the sensing volume. After this period, the buffer-baseline returned indicating the reversibility of the assays. The data indicated that responses to at least two basic channel-regulating drugs were preserved in the experimental set-up.

The effects of ion channel modifying pharmaceuticals have been studied mainly by electrophysiological methods using living cells, tissue slices or experimental animals. For screening purposes, however, less time-consuming and more standardized *in vitro* models would be ideal. The assay system we propose is based on three ideas: i) functions of membrane-embedded ion channel and transporter assemblies can be monitored if refractive index changes caused by migration of transported compounds are detected in a small, *separated sensing volume*; ii) evaluable OWLS records can be get even if the *fluid compartments are not completely insulated*; iii) in lipid-filled filter-membranes, the functions of transmembrane molecule complexes can be investigated *without isolation and subsequent reconstitution of membrane proteins* in artificial lipid environment.

Our data showed that native biomembrane fragments can be inserted into porous filter-sheets with preservation of natural channel activity. The membrane-filled filter-sheets, besides serving as object holders, can insulate fluid compartments. Insulator functions can be reached and/or improved if “empty” filter pores in the holder membrane are filled up with liposome-derived lipid material, and the sensing volume are kept free from lipids by a thin, water-permeable separating membrane. The presented data indicate that ion-drifts in a small sensing volume can provide well detectable endpoints for optical detection of opening and closing of ion channels. Compartmentalized OWLS sensor-assemblies provide sensitive optical detection in a small, separated sensing volume, and support membrane-embedded proteins. Preservation of GABA-gated channel activity and recording responses to agonist and antagonist drugs encourage further development of membrane-sandwich sensor set-ups for *in vitro* pharmacological studies by optical methods.

## Conclusions

By measuring ion-drifts in a small sensing volume, compartmentalized OWLS sensor-assemblies allowed detecting functions of ion channels supported by lipid-filled commercial membranes. Lipid material was filled in a fibrous holder lattice, which did not prevent the self-assembly of gramicidin channels, and preserved some functional characteristics of biomembrane-embedded, cell-derived GABA_A_ receptors. The sensing volume was kept free from lipid material by inserting a thin water- and ion-permeable separator membrane. The compartmentalized sensor assembly provided:

- satisfactory ion-insulation, which was modulated by insertion and/or opening of ion channels;
- an easily fabricated environment for functional assembly of membrane-associated ion channels even with large molecular weight and multi-unit composition;
- a simple principle for developing sensor set-ups for sensitive optical detection of functional responses of biomembrane-embedded drug-targets.

## Supporting Information

File S1 **Combined Supporting Information S1.** S1.1. Preparation of liposomes. S1.2. Lipid coverage of the holder membrane. S1.3. Expression of GABAA^α5β2γ2^ receptor by HEK293 cells. S1.4. Assays on H_2_O/D_2_O exchange using liposomes immobilized directly on the sensor surface. S1.5. Texas Red labeled DNA-cross-linked liposomes on the sensor surface. S1.6. Time-course of washing out of ethanolamine from liposomes prepared in Tris-buffered ethanolamine.
(DOC)

## References

[1] Homola J (2006) Surface plasmon resonance based sensors. Springer-Verlag, Berlin-Heidelberg-New York. 251 p.

[2] Vörös J, Ramsden JJ, Csúcs G, Szendrő I, De Paul SM, at al (2002) Optical grating coupler biosensors. Biomaterials 23: 3699–3710. 12109695 10.1016/s0142-9612(02)00103-5

[3] Bally M, Bailey K, Sugihara K, Grieshaber D, Vörös J, at al (2010) Liposome and bilayer arrays towards biosensing applications. Small 6: 2481–2497. 20925039 10.1002/smll.201000644

[4] Heimburg T (2010) Lipid ion channels. Biophys Chem 150: 2–22. 20385440 10.1016/j.bpc.2010.02.018

[5] Majd S, Yusko EC, Billeh YN, Macrae MX, Yang J, at al (2010) Applications of biological pores in nanomedicine, sensing, and nanoelectronics. Curr Opin Biotechnol 21: 439–476. 20561776 10.1016/j.copbio.2010.05.002 PMC3121537

[6] Mashaghi A, Swann M, Popplewell J, Textor M, Reimhult E (2008) Optical anisotropy of supported lipid structures probed by waveguide spectroscopy and its application to study of supported lipid bilayer formation kinetics. Anal Chem 80: 3666–3676. 18422336 10.1021/ac800027s

[7] Baumann MK, Swann MJ, Textor M, Reimhult E (2011) Pleckstrin homology-phospholipase C-δ_1_ interaction with phophatidylinositol 4,5-biphosphate containing supported lipid bilayers monitored in situ with dual polarization interferometry. Anal Chem 83: 6267–6274. 21728304 10.1021/ac2009178

[8] Steller L, Kreir M, Salzer R (2012) Natural and artificial ion channels for biosensing platforms. Anal Bioanal Chem 402: 209–230. 22080413 10.1007/s00216-011-5517-y

[9] Tiefenthaler K (1992) Integrated optical couplers as chemical waveguide sensors. Adv Biosens 2: 261–289.

[10] Ramsden JJ (1993) Review of new experimental techniques for investigating random sequential adsorption. J Stat Phys 73: 853–877.

[11] Erdélyi K, Frutos AG, Ramsden JJ, Szendrő I, Voirin G (2007) In: Marks RS, Cullen DC, Karube I, Lowe CR, Weetall HH, editors. Handbook of biosensors and biochips. Wiley, Chichester. 569–586.

[12] MicroVacuum website. Available: http//www.owls-sensors.com. Accessed 2013 Oct 25.

[13] Sackmann E (1996) Supported membranes: scientific and practical applications. Science 271: 43–48. 8539599 10.1126/science.271.5245.43

[14] Kügler R, Knoll W (2002) Polyelectrolyte-supported lipid membranes. Bioelectrochemistry 56: 175–178. 12009469 10.1016/s1567-5394(02)00031-2

[15] Sugihara K, Vörös J, Zambelli T (2010) A gigaseal obtained with a self-assembled long-lifetime lipid bilayer on a single polyelectrolyte multilayer-filled nanopore. ACS Nano 4: 5047–5054. 20687537 10.1021/nn100773q

[16] Phung T, Zhang Y, Dunlop J, Dalziel J (2011) Bilayer lipid membranes supported on Teflon filters: a functional environment for ion channels. Biosens Bioelectron 26: 3127–35. 21211957 10.1016/j.bios.2010.12.013

[17] Sigel E, Steinmann ME (2012) Structure, function and modulation of GABA_A_ receptors. J Biol Chem Doi: 10.1074/jbc.R112.386664 10.1074/jbc.R112.386664 PMC3504738 23038269

[18] Moscho A, Orwar O, Chiu DT, Modi BP, Zare RN (1996) Rapid preparation of giant unilamellar vesicles. Proc Natl Acad Sci USA 93: 11443–11447. 8876154 10.1073/pnas.93.21.11443 PMC38076

[19] Trummer N, Adányi N, Váradi M, Szendrő I (2001) Modification of the surface of integrated optical wave-guide sensors for immunosensor applications. Fresenius J Anal Chem 371: 21–24. 11605752 10.1007/s002160100929

[20] Nic M, Jirat J, Kosata B (2006–) “Limit of detection”. IUPAC Compendium of Chemical Terminology (Online ed.) Doi:10.1351/goldbook.L03540.

[21] Brändén M, Dahlin S, Höök F (2008) Label-free measurements of molecular transport across liposome membranes using evanescent-wave sensing. Chemphyschem 9: 2480–2485. 19034923 10.1002/cphc.200800614

[22] Brändén M, Tabaei SR, Fischer G, Neutze R, Höök F (2010) Refractive-index-based screening of membrane-protein-mediated transfer across biological membranes. Biophys J 99: 124–133. 20655840 10.1016/j.bpj.2010.03.059 PMC2895391

[23] Hemsley G, Busath D (1991) Small iminium ions block gramicidin channel in lipid bilayers. Biophys J 59: 901–907. 1712240 10.1016/S0006-3495(91)82303-7 PMC1281256

[24] Horváth R, Vörös J, Graf R, Fricsovszky G, Textor M, et al (2001) Effect of patterns and inhomogeneities on the surface of waveguides used for optical waveguide lightmode spectroscopy applications. Appl Phys B: Lasers Opt 72: 441–447.

[25] Cottier K, Horvath R (2008) Imageless microscopy of surface patterns using optical waveguides. Appl Phys B: Lasers Opt 91: 319–327.

[26] Kelkar DA, Chattopadhyay A (2007) The gramicidin ion channel: a model membrane protein. Biochim Biophys Acta 1768: 2011–2025. 17572379 10.1016/j.bbamem.2007.05.011

[27] Birnir B, Eghbali M, Cox GB, Gage PW (2001) GABA concentration sets the conductance of delayed GABAA channels in outside-out patches from rat hippocampal neurons. J. Membrane Biol 181: 171–183. 11420604 10.1007/s00232-001-0021-5

