# Supplementary material for: Optical Waveguide Lightmode Spectroscopic Techniques for Investigating Membrane-Bound Ion Channel Activities
Source: PLoS One. 2013 Dec 10;8(12):e81398. doi: 10.1371/journal.pone.0081398 (PMC3858217; doi:10.1371/journal.pone.0081398)
Supplement: File S1 — Combined Supporting Information S1. S1.1. Preparation of liposomes. S1.2. Lipid coverage of the holder membrane. S1.3. Expression of GABAAα5β2γ2 receptor by HEK293 cells. S1.4. Assays on H2O/D2O exchange using liposomes immobilized directly on the sensor surface. S1.5. Texas Red labeled DNA-cross-linked liposomes on the sensor surface. S1.6. Time-course of washing out of ethanolamine from liposomes prepared in Tris-buffered ethanolamine. (DOC) [file pone.0081398.s001.doc]

**Combined Supporting Information S1**

**Optical Waveguide Lightmode Spectroscopic Techniques for Investigating Membrane-Bound Ion Channel Activities**

Inna Székács^*^, Nóra Kaszás, Pál Gróf, Katalin Erdélyi, István Szendrő, Balázs Mihalik, Ágnes Pataki, Ferenc A. Antoni, Emilia Madarász

* Institute of Experimental Medicine, Hungarian Academy of Sciences

Szigony u. 43, H-1083 Budapest, Hungary

E-mail: inna.szekacs@gmail.com

***S1.1.*** *Preparation of liposomes*

Stock solution of 10 mg/ml egg yolk lecithin (composition: 70% phosphatidylcholine, 10% phosphatidylethanolamine and 20% other lipids including neutral lipids) was prepared in chloroform-methanol (9:1) mixture. 2 ml of stock solutions containing 2-4 mg lecithin were mixed with Texas Red (molar ratio 3000:1) and/or with DOPE-biotin (molar ratio 4%). Briefly, 2 ml lipid mixture was pipetted into a 100 ml round-bottomed flask of a Buchi rotavapor device (BÜCHI AG, Switzerland) immersed into a 30^o^C water-bath. 7 ml of corresponding buffer was layered above the organic solution and the organic solvent was removed from the rotating flask by an oil-vacuum pump (final vacuum <20 Hgmm). The remaining turbid suspension (~6 ml) was dispensed in 1.5 ml Eppendorf tubes, centrifuged at 2085 g for 10 min. Pellets were collected in 600 µl buffered saline. Quality of the liposome preparation was checked by phase-contrast or confocal fluorescence microscope and was accepted for further use if the proportion of multilamellar structures was negligible. Mixed population of liposomes contained mainly large (LUV) and giant (GUV) unilamellar vesicles.

***S1.2.*** *Lipid coverage of the holder membrane*

**
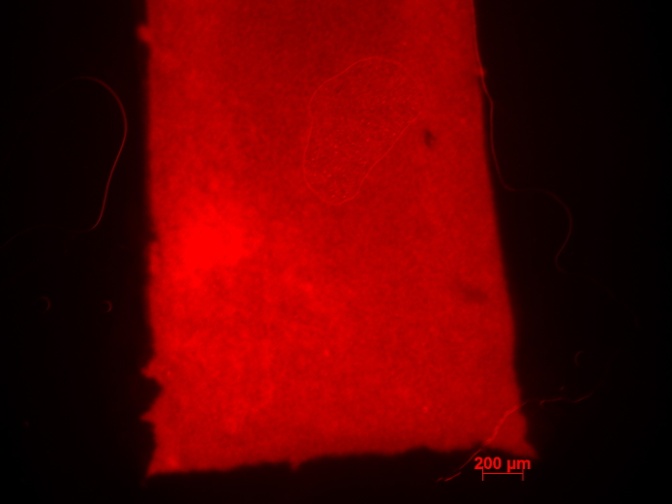
**

**Figure A in File S1.** View of the PTFE holder membrane with settled lipid material derived from Texas Red labeled liposomes at the end of the experimental procedures

***S1.3.*** *Expression of GABA_A_^α5β2γ2^ receptor by HEK293 cells*

The expression of GABA_A_ receptor subunits was checked by staining with anti-α5 (mouse IgG; 1/500; Chemicon) and anti-β2 antibodies (rabbit IgG; 1/1000; Chemicon) after fixation with 4% (w/v) paraformaldehyde in PBS (pH 7.4) and was visualized with Alexa-594 and Alexa-488 labeled anti-mouse and anti-rabbit secondary antibodies (1/2000), respectively (Figure B in File S1).


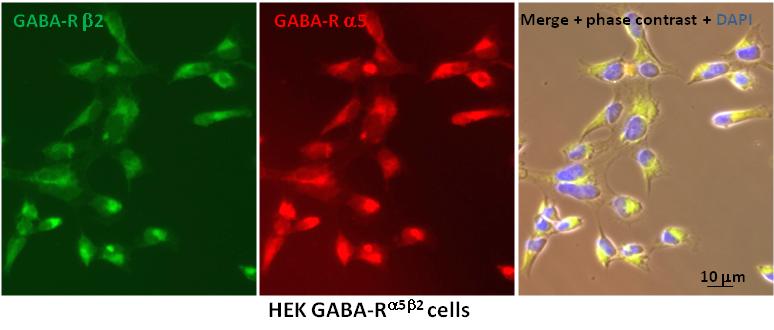


**Figure B in File S1.** Immunocytochemical demonstration of the expression GABA_A_β2 (green) and GABA_A_α5 (red) receptor subunits in HEK293 cells

***S1.4.*** *Assays on H_2_O/D_2_O exchange using liposomes immobilized directly on the sensor surface*

Considering the significantly lower refractive index of D_2_O in comparison to H_2_O (Table 1 in the main text), the rate of H_2_O/D_2_O exchange was monitored in the presence and absence of gramicidin channels. Monovalent cations get through these channels and are followed with water; therefore the move of water provides a measure of channel opening. Liposomes containing both DOPE-biotin and Texas Red were prepared in H_2_O-based TBS (H_2_O-TBS) and anchored to NeutrAvidin-coated sensor surfaces. Settled liposomes were washed with H_2_O-TBS under continuous OWLS recording. After reaching a stable baseline, 100 µl of D_2_O-based TBS (D_2_O-TBS) was streamed through the cuvette followed by H_2_O-TBS washing (Figure C in File S1). The injection-washing cycles were repeated three-times with continuous flow-rate (23 µl/min), then gramicidin was added and the H_2_O/D_2_O exchange-assays were repeated with three subsequent injections of D_2_O-TBS.

Regardless of gramicidin channels, the effective refractive indices (NTE and NTM) decreased rapidly in response to D_2_O-TBS (Figure C in File S1). The intense response in the absence of ion channels indicated an important proportion of free diffusion neglecting the lipid barriers. The rapid diffusion might be resulted by discontinuities in the lipid/liposome coverage even if fluorescence microscopy revealed uniform stain distribution. Also, a considerable rate of H_2_O/D_2_O exchange through channel-free liposome membranes (Tredgold and Jones, 1979; Mamonov et al., 2007) could not be excluded. In the presence of gramicidin channels the amplitude of changes failed to show measurable differences, but the optical changes were delayed (Figure C insert in File S1). The extended time-course indicated that H_2_O/D_2_O-exchange slowed down in at least a fraction of the available reaction volume. As a plausible explanation, intra-liposome space might be opened up by gramicidin channels and a slower H_2_O/D_2_O-exchange through the channels might cause the delay in the equilibration kinetics (Chiu et al., 1999).

As the magnitude of any optical changes was far beyond that would be required for a stable and reproducible assay, detailed analyses and further efforts were not performed to develop that sort of membrane permeability assays.

**Figure C in File S1.** OWLS detection on H_2_O/D_2_O exchange (**5**) before and after incorporating gramicidin (**6**) into liposomes attached to the sensor surface by NeutrAvidin–biotin binding. The surface was treated with 2.5% glutaraldehyde in water (**1**), then 40 µg/ml of NeutrAvidin in water was added (**2**). The surface was then equilibrated with H_2_O-TBS (**3**), and biotinylated liposomes hydrated with H_2_O-TBS were let to bind (**4**) for 40 min without buffer flow. After washing with H_2_O-TBS, 100 µl aliquots of D_2_O-TBS (**5**) were streamed through the cuvette (23 µl/min) followed by washing with H_2_O-TBS until reaching a stable baseline. The procedure was repeated 3-times before and after gramicidin (**6**) incorporation. The main graph shows the changes in the effective refractive index recorded in the transverse magnetic mode (NTM). The insert shows changes of the refractive index (n_cTM_ measured in the transverse magnetic mode) on the sensor surface during D_2_O-TBS injections before (○) and after (●) gramicidin incorporation. Zero time-point indicates the starting time of each injection

**References**

Chiu SW, Subramaniam S, Jakobsson E (1999) Biophys J 76: 1939–50.

Mamonov AB, Coalson RD, Zeidel ML, Mathai JC (2007) J Gen Physiol 130: 111–116.

Tredgold RH, Jones R (1979) Biochim Biophys Acta 550: 543–545.

***S1.5.*** *Texas Red labeled DNA-cross-linked liposomes on the sensor surface*


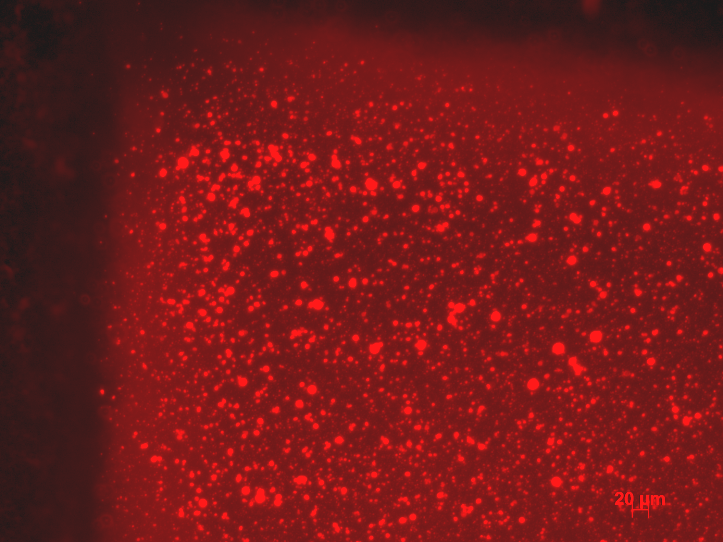


**Figure D in File S1.** Texas Red labeled DNA-cross-linked liposomes stayed stably on the sensor surface by the end of the experimental procedures

***S1.6.*** *Time-course of washing out of ethanolamine from liposomes prepared in Tris-buffered ethanolamine*

In another series of experiments, liposomes hydrated with 141.5 mM ethanolamine in 25 mM Tris (pH 7.4) were attached to the holder membrane, and ethanolamine washing out was assayed before and after the incorporation of gramicidin channels. Liposomes attached to the PTFE filter membrane were perfused with ethanolamine-Tris buffer until reaching a stable baseline, then 100 µl TBS (141.5 mM NaCl; 25 mM Tris; pH 7.4) was injected. The time-course of ethanolamine washing out was indicated by a decrease of the effective refractive indices (NTE, NTM). In the presence of gramicidin channels, ethanolamine was removed at an about 2-fold higher rate in comparison to the clearance from liposomes without channels (Figure E in File S1).


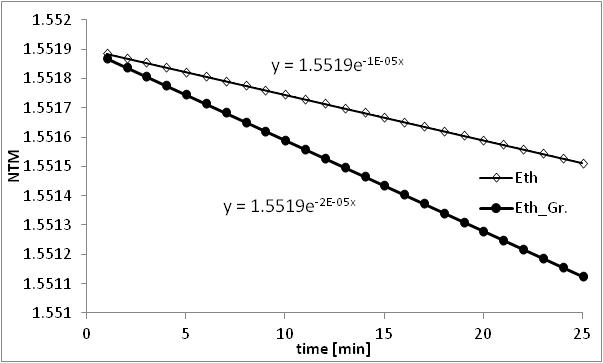


**Figure E in File S1.** Time-course of washing out of ethanolamine through the membrane-sandwich with (●) or without (○) gramicidin treatment, calculated from the first 6 minutes of washing out period in a representative experiment. Liposomes were prepared in 141.5 mM ethanolamine in 25 mM Tris, and were perfused with 141.5 mM NaCl buffered with 25 mM Tris
